# Non-heme iron overload impairs monocyte to macrophage differentiation *via* mitochondrial oxidative stress

**DOI:** 10.3389/fimmu.2022.998059

**Published:** 2022-10-21

**Authors:** Yue Cui, Saray Gutierrez, Sheller Ariai, Lisa Öberg, Kristofer Thörn, Ulf Gehrmann, Suzanne M. Cloonan, Thomas Naessens, Henric Olsson

**Affiliations:** ^1^ Translational Science & Experimental Medicine, Research and Early Development, Respiratory & Immunology, BioPharmaceuticals R&D, AstraZeneca, Gothenburg, Sweden; ^2^ Bioscience Cardiovascular, Early Cardiovascular, Renal and Metabolism (CVRM), BioPharmaceuticals R&D, AstraZeneca, Gothenburg, Sweden; ^3^ Early Product Development, Pharmaceutical Sciences, BioPharmaceuticals R&D, AstraZeneca, Gothenburg, Sweden; ^4^ Division of Pulmonary and Critical Care Medicine, Joan and Sanford I. Weill Department of Medicine, Weill Cornell Medical College, New York, NY, United States; ^5^ School of Medicine, Trinity Biomedical Sciences Institute and Tallaght University Hospital, Trinity College Dublin, Dublin, Ireland; ^6^ Bioscience Cough & In vivo, Research and Early Development, Respiratory & Immunology, BioPharmaceuticals R&D, AstraZeneca, Gothenburg, Sweden

**Keywords:** iron, differentiation, macrophage, mitochondria, heme, oxidative stress, MAFB, COPD

## Abstract

Iron is a key element for systemic oxygen delivery and cellular energy metabolism. Thus regulation of systemic and local iron metabolism is key for maintaining energy homeostasis. Significant changes in iron levels due to malnutrition or hemorrhage, have been associated with several diseases such as hemochromatosis, liver cirrhosis and COPD. Macrophages are key cells in regulating iron levels in tissues as they sequester excess iron. How iron overload affects macrophage differentiation and function remains a subject of debate. Here we used an *in vitro* model of monocyte-to-macrophage differentiation to study the effect of iron overload on macrophage function. We found that providing excess iron as soluble ferric ammonium citrate (FAC) rather than as heme-iron complexes derived from stressed red blood cells (sRBC) interferes with macrophage differentiation and phagocytosis. Impaired macrophage differentiation coincided with increased expression of oxidative stress-related genes. Addition of FAC also led to increased levels of cellular and mitochondrial reactive oxygen species (ROS) and interfered with mitochondrial function and ATP generation. The effects of iron overload were reproduced by the mitochondrial ROS-inducer rotenone while treatment with the ROS-scavenger N-Acetylcysteine partially reversed FAC-induced effects. Finally, we found that iron-induced oxidative stress interfered with upregulation of M-CSFR and MAFB, two crucial determinants of macrophage differentiation and function. In summary, our findings suggest that high levels of non-heme iron interfere with macrophage differentiation by inducing mitochondrial oxidative stress. These findings might be important to consider in the context of diseases like chronic obstructive pulmonary disease (COPD) where both iron overload and defective macrophage function have been suggested to play a role in disease pathogenesis.

## Introduction

Macrophages are tissue-resident innate immune cells that play an important role in host defense, tissue homeostasis and repair, and inflammatory responses (1). Macrophages are highly plastic and adaptable to signals from the tissue niche (2). In steady-state, macrophages originate either from embryonic precursors or from circulating monocytes (3, 4). In response to inflammation or tissue injury, circulating monocytes migrate to tissues and differentiate locally into macrophages. They play an important role in shaping the inflammatory response and its resolution in tissues (3, 5–7). In humans, the two main subsets of circulating monocytes are CD14^+^CD16^-^ classical monocytes and CD14^lo^CD16^+^ non-classical monocytes. Classical monocytes are known for their potential to migrate to inflamed or injured tissues (8–10).

Iron is a nutrient element that is essential in many biological processes, including DNA synthesis, cellular energy production, metabolic enzyme activity, and immune function and metabolism (11–13). Systemic and cellular iron homeostasis are tightly regulated with finely tuned iron regulatory mechanisms. An excess of intracellular iron is detrimental as it induces oxidative stress, lipid peroxidation, DNA damage, and mitochondrial dysfunction (11, 14).

Macrophages are the principal immune cells responsible for iron handling and vital for systemic iron homeostasis (15). Conversely, iron shapes macrophage polarization toward an M1 proinflammatory phenotype in Lewis lung carcinoma (16) and spinal cord injury models (17) or a pro-resolution M2 phenotype in diabetic wound healing in mice (18) and THP-1 monocyte-derived macrophages under chronic iron overload (19).

Many disorders, including hemochromatosis, metabolic disorders, infectious diseases, and chronic obstructive pulmonary disease (COPD) (20–24), are associated with a perturbation of iron metabolism, resulting in systemic or local iron overload (25). Given that circulating monocytes can migrate and differentiate locally into macrophages, iron overload may have distinct functional consequences on monocyte and macrophage populations. For instance, iron-accumulation in macrophages and macrophage dysfunction have been associated with COPD, and it has been suggested that iron overload in macrophages may be a contributing factor to COPD disease pathogenesis (21, 26–30). Severe alcoholic hepatitis is also associated with iron accumulation in hepatocytes/macrophages, and it has been suggested that iron-mediated inflammation and macrophage activation could be inhibited with iron chelation (23).

Here, we used an *in vitro* model to understand the functional effect of iron overload on macrophage differentiation and function. Our study shows that iron overload induces aberrant monocyte-to-macrophage differentiation resulting in impaired macrophage function. These iron-induced phenotypic and functional changes are in part due to iron-induced oxidative stress and mitochondrial dysfunction since treatment with ROS scavenger alleviates iron-induced defective monocyte-to-macrophage differentiation. Overall, our study provides a mechanistic insight into the effects of iron overload on monocyte-to-macrophage differentiation and their possible implications in iron-induced pathophysiology.

## Materials and methods

### Subject and samples

All subjects provided informed written consent for blood donation as approved by AstraZeneca’s Institutional review board and local ethic committee (033–10). Heparin-anticoagulated whole blood samples were collected from healthy donors and processed on the same day of collection.

### Monocyte isolation and culture

Peripheral blood mononuclear cells (PBMC) were isolated from whole blood by density centrifugation using Lymphoprep (STEMCELL Technologies) or Ficoll-Paque (GE Healthcare). Monocytes were isolated from PBMCs using Classical Monocyte Isolation Kit (Miltenyi Biotec) or Monocyte Isolation kit (STEMCELL Technologies) according to the manufacturer’s instructions. Monocytes were differentiated for 3-5 days in X-Vivo 15 media (Lonza) supplemented with 4mM L-glutamine, 100 U/mL penicillin/streptomycin (Thermo Fisher Scientific), and 100 ng/mL M-CSF (PeproTech) to generate monocyte-derived macrophages (MDMs).

### Preparation of stressed red blood cells

Stressed red blood cells were obtained using a previously described protocol with modification (5). Blood was collected and centrifuged at 500 RCF for 10 min. After removal of buffy coat, RBCs were washed twice with PBS. sRBCs were generated by shaking at 48°C for 20 min. sRBC were added to monocytes in a 10:1 ratio (10 sRBC per monocyte).

### Chemicals and drugs

The following chemicals were purchased from Sigma: ferric ammonium citrate (FAC, F5879), Deferoxamine mesylate salt (DFO, D9533), N-Acetyl-L-cysteine (NAC, A9165), Hemin (51280). The FAC stock solution was prepared at 100 mg/mL (381.72 mM) in H_2_O with 17.6 mg Fe/mL. The hemin stock solution was prepared at 40mM in 0.15M NaCl containing 1.4M NH_4_OH. The stock solution of DFO and NAC were prepared in DMSO at 87.5mM and 1M respectively. Oligomycin, rotenone, FCCP and Antimycin A were purchased from Agilent as part of the Seahorse XF Cell Mito Stress Test Kit and prepared following manufacturer’s instructions.

### Flow cytometry

Flow cytometry was performed with directly conjugated antibodies or fluorescent probes according to standard techniques and analyzed on Fortessa flow cytometers (BD). The antibody clones used included: MERTK-PE-Cy7 (BioLegend, clone 590H11G1E3), CD206-AlexaFluor700 (BioLegend, clone 15-2), CD71-BV711 (BD Biosciences, clone M-A712), CD115-AlexaFluor647 (BD Biosciences, clone 9-4D2-1E4), Ferroportin-PE (Novus Biologicals, clone 8G10NB), CD86-PerCP-Cy5.5 (BD Biosciences, clone 2331 (FUN-1)), CD14-BV421 (BioLegend, clone M5E2), CD11b-FITC (BioLegend, clone ICRF44), HLA-DR-APC (BioLegend, clone L243), CD16-BV786 (BD Biosciences, clone 3G8). All samples were incubated with Fc block (BD Biosciences or BioLegend) for 15 minutes at 4°C prior to incubation with antibodies for 20 minutes at 4°C. 7-AAD (Life Technologies), Zombie Green Fixable Viability Kit (BioLegend, Ex/Em=488/515 nm) and Fixable Viability Dye eFluor 780 (eBioscience, Ex/Em=633/780 nm) were used to exclude dead cells. Annexin V was used to detect apoptotic cells. Cellular ROS was measured using CellROX Green reagent (Invitrogen, Ex/Em=485/520 nm). CellROX (5 µM) was added directly into the cell culture medium and incubated for 30 min at 37°C prior to FACS analysis. Mitochondrial ROS was measured using MitoSOX Red mitochondrial superoxide indicator (Invitrogen, Ex/Em=510/580 nm). MitoSOX was used at a final concentration of 5µM, and samples were incubated for 10 minutes at 37°C. Mitochondrial membrane potential was measured using MitoProbeTMRM kit (Invitrogen, Ex/Em=550/561 nm) following manufacturer’s instructions. Mitochondrial mass was assessed by incubating cells with MitoTracker Deep Red FM (Invitrogen, Ex/Em=644/665 nm) at a concentration of 50nM for 30 minutes at 37°C. Data was analyzed with FlowJo (BD).

### Phagocytosis assay

MDMs culture medium was replaced with live cell imaging solution containing 100 µg/mL pHrodo Red or 50 µg/mL Green *E.coli* Bioparticles (Invitrogen) for 1h at 37°C. After incubation, cells were washed extensively prior to phagocytic activity measurement by flow cytometry. Viability dye was added to exclude dead cells from the analysis. Incubation at 4°C was used as a negative control.

### Metabolic extracellular flux analysis

Oxygen consumption rate (OCR) was assessed in MDMs using the Seahorse XFe Analyzer (Agilent) and the Seahorse XF Cell Mito Stress Test Kit (Agilent) according to manufacturer’s instructions. Final drug concentrations used were 0.5µM for Oligomycin and Antimycin A, and 1µM for oligomycin and FCCP.

### mRNA-Seq and analysis

RNA was isolated with the RNeasy Plus 96 kit (Qiagen). Libraries for RNA-seq were generated using TruSeq Stranded mRNA kit (Illumina) with dual indexing adaptors. Libraries were validated on the Fragment Analyzer platform (AATI) and concentrations were determined using the Quant-iT dsDNA HS assay kit on the Qubit fluorometer (ThermoFisher scientific). Sample libraries were pooled in equimolar concentrations, diluted, and denatured according to Illumina guidelines. Sequencing was performed using a High Output flow cell on an Illumina NextSeq500. RNA-seq fastq files were processed using bcbio-nextgen (v.1.1.6a-b’2684d25’) (https://github.com/chapmanb/bcbio-nextgen ) where reads were mapped to the human genome build hg38 using hisat2 (31) (v.2.1.0) yielding between 3.7-12.5 M reads (6.4 M on average) with a 96% mapping frequency or higher per sample. Library and sequencing quality was assessed using fastqc (v.0.11.8) (http://www.bioinformatics.babraham.ac.uk/projects/fastqc/ ), qualimap (32) (v.2.2.2c), and samtools (33) (v.1.9), summarized using MultiQC (34) (v1.7). Gene level quantifications were generated with Salmon (35) (v.0.14.1) within bcbio-nextgen. OmicSoft Studio software (version 10, Qiagen OmicSoft) was used for further data analysis. Differential gene expression was assessed with DESeq2 (36). Genes were considered significantly differentially expressed if they had a q<0.05 with Benjamini-Hochberg multiple correction. Bioinformatic analysis for canonical signaling pathways and upstream regulators was performed using Ingenuity Pathway Analysis software (Qiagen).

### RNA isolation and quantitative PCR

RNA was isolated with the RNeasy Plus Mini or Micro kit (Qiagen), followed by cDNA transcription with High Capacity cDNA Reverse Transcription Kit (Applied Biosystems). Real-time PCR was performed on a QuantStudio 7 Flex Real-Time PCR System (Applied Biosystems). Taqman Gene Expression Assays MAFB (Hs00534343_s1), CSF1R (Hs00911250_m1), HMOX1 (Hs01110250_m1), GCLM (Hs00978072_m1), NQO1 (Hs01045993_g1), and Taqman Fast Advanced Master Mix (Applied Biosystems) were used according to the manufacturer’s instructions. Fold change in expression was determined by the 2-ΔΔCT method after normalizing to GAPDH (Hs02786624_g1) and B2M (Hs00187842_m1).

### Cytokine and chemokine secretion

MDMs were stimulated with 10 ng/mL Ultrapure LPS (*In vivo*gen) or 1 µg/mL CD40L oligomer (Enzo Life Sciences) for 24h. Supernatants were collected and cytokine/chemokine were quantified using MSD U-PLEX kit (Meso Scale Diagnostics).

### Iron quantification

Cell pellet was dissolved in 2mL 20% HNO_3_, digested in Microwave digestion system for 30 minutes, and diluted to 2.5 ml in final volume for analysis. 0.01, 0.1 and, 1 μg/ml Fe in 20% HNO_3_ were used as calibration standards, 0.2 μg/ml Indium was used as internal standard. Cellular iron was quantified using inductively coupled plasma-mass spectrometry (ICP-MS, Agilent 7900).

### ATP quantification

ATP was quantified using the CellTiter-Glo^®^ 2.0 kit (Promega) following manufacturer’s instructions.

### Statistics

All data were analyzed with one-way or two-way ANOVA with Dunnett’s multiple comparisons or Friedman test with Dunn’s multiple comparisons test using GraphPad Prism 8 (GraphPad Software Inc.). Data were considered significant at a P value of less than 0.05. All data are reported as the median, arithmetic or geometric mean ± SD as appropriate.

## Results

### Iron overload alters phenotype and function of monocyte-derived macrophages

To investigate the role of iron overload in monocyte-to-macrophage differentiation, we established an *in vitro* model. Specifically, heme iron (stressed red blood cells, sRBC, or hemin) or non-heme iron (ferric ammonium citrate, FAC) was used as iron sources in peripheral blood monocyte cell cultures in presence of macrophage colony stimulating factor (M-CSF) to induce differentiation into macrophages. Adding FAC and sRBC, but not hemin, led to iron accumulation in monocyte-derived macrophages (MDM), as demonstrated by inductively coupled plasma-mass spectrometry analysis (**Figure S1**).

**Figure 1:**
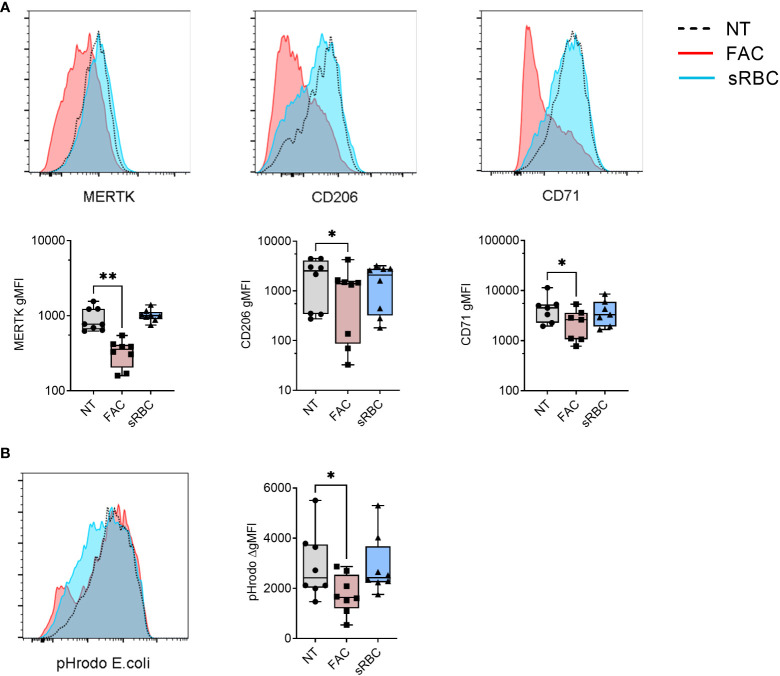
Iron overload by FAC, but not sRBC, prevents M-CSF-stimulated monocyte to macrophage differentiation. **(A)** Surface expression of indicated markers analyzed by FACS on monocyte-derived macrophages differentiated with M-CSF in the presence of ferric ammonium citrate (FAC, 4 µg Fe/mL), stressed RBC (sRBC, 10x monocytes) or with M-CSF alone (NT) for 5 days. Each symbol represents an individual donor (n = 7-8). Data shown are pooled from three experiments. **(B)** Phagocytic capacity of macrophages differentiated with M-CSF in the presence of FAC (4 µg Fe/mL) or sRBC, quantified as the increment in fluorescence intensity of engulfed pHrodo *E. coli* bioparticles between cells incubated at 37°C and at 4°C. Each dot represents an individual donor (n = 8). Data shown are pooled from two experiments. **(A, B)** One-way ANOVA with Dunnett’s multiple comparisons test was performed. *p < 0.05, **p < 0.01.

Next, we examined the phenotype of MDMs differentiated in the presence of FAC or sRBC by analyzing the MDM surface maturation markers MER Proto-Oncogene Tyrosine Kinase (MERTK) (37), mannose receptor C type 1 (CD206) (38), and transferrin receptor 1 (CD71) (39). After 5 days of culture, FAC inhibited M-CSF-stimulated surface expression of MERTK, CD206, and CD71 by about 50% whereas exposure to sRBC did not alter surface expression levels of any of these markers (**Figure 1A**). This effect was observed as early as day 1 of monocyte differentiation with M-CSF (**Figure S2A**). In contrast, FAC did not alter the surface expression of the iron exporter ferroportin 1 (FPN1) or the MDM markers CD86, CD14, HLA-DR, but it slightly decreased surface expression of CD11b and CD16 compared to monocytes not exposed to additional iron (**Figure S2B**). MDM differentiated in the presence of sRBC exhibited a phenotype of iron recycling macrophages (5) with an increased surface expression of FPN1, CD14, CD86, and CD11b (**Figure S2B**).

In accordance with their less mature phenotype with reduced surface expression of MERTK, CD206, and CD71, MDM differentiated in the presence of FAC showed decreased capacity to phagocytose pHrodo-coated *E.coli* Bioparticles (**Figure 1B**) and reduced cytokine (IL-10, IL-23, IL-8) and chemokine (CCL2) production in response to stimulation with LPS or CD40L compared to monocytes differentiated without the addition of external iron (**Figure S3**). MDMs differentiated with M-CSF in the presence of sRBC displayed no significant changes in phagocytic function or ability to respond to LPS or CD40L.

The observed changes in MDM phenotype and function caused by exposure to FAC or sRBC were not associated with increased apoptosis, as we observed a similar percentage of Annexin V+ 7-AAD+ cells in MDM differentiated in the presence of FAC or sRBC compared to control conditions (**Figures S4A, S4B**).

Overall, the above results suggest that non-heme iron overload inhibits monocyte-to-macrophage differentiation *in vitro.*

### Iron overload induces oxidative stress responses and mitochondrial dysfunction

**Figure 2:**
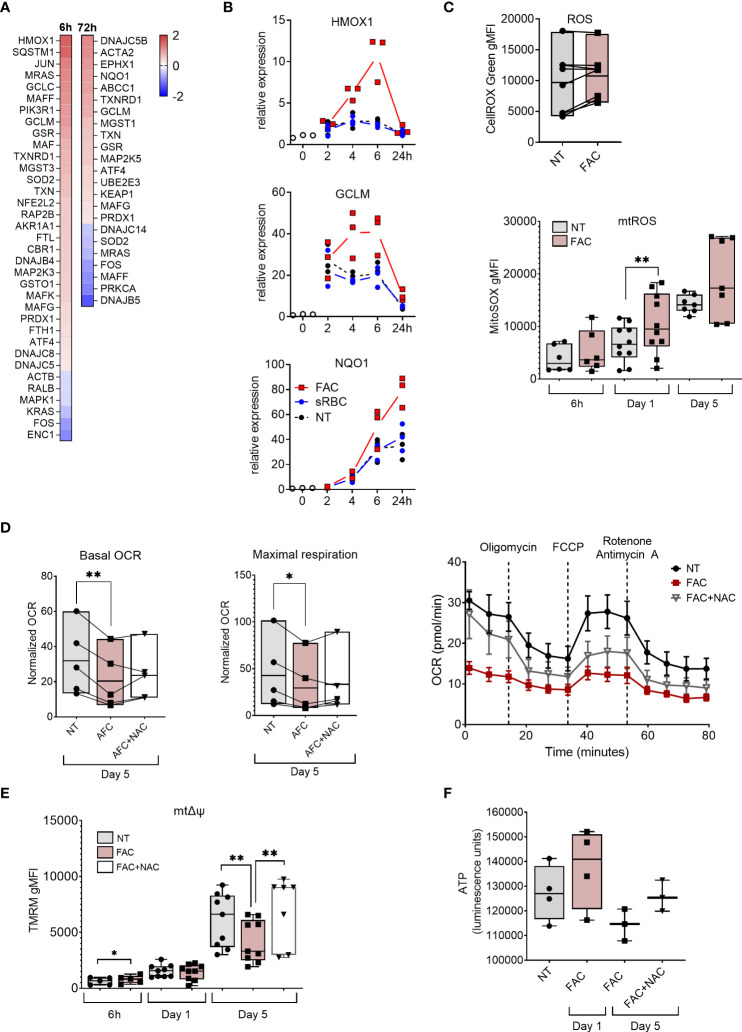
Iron overload upregulates oxidative stress response genes and induces mtROS production and mitochondrial dysfunction. **(A)** Heatmap depicting Log2 fold change of NRF2-mediated oxidative stress response genes in monocytes cultured with M-CSF and FAC (4 µg Fe/mL) for 6h or 72h compared to monocytes cultured with only M-CSF **(B)** mRNA expression relative to 0h of NRF2-regulated genes in monocytes cultured with M-CSF and FAC (4 µg Fe/mL) or sRBC for 0-24 h. **(C)** FACS analysis of cellular ROS at day 5 (n = 8) and of mtROS production (n = 6-10) at indicated time points, in monocytes differentiated with M-CSF in the presence of FAC (4 µg Fe/mL) **(D)** Basal oxygen consumption rate (OCR) and maximal respiration in monocytes differentiated with M-CSF in the presence of FAC (4 µg Fe/mL) for 5 days. Cells were also treated with a combination of FAC (4 µg Fe/mL) and N-acetyl-L-cysteine (NAC, 4mM) for 5 days. A representative OCR analysis of monocytes from one single donor is also shown. **(E)** FACS analysis of mitochondrial membrane potential (mΔψ) in monocytes differentiated with M-CSF in the presence of FAC (4 µg Fe/mL) for 6h, 1 day and 5 days or a combination of FAC (4 µg Fe/mL) and NAC (4mM) for 5 days (n = 6-8). **(F)** ATP quantification in monocytes differentiated with M-CSF in the presence of FAC (4 µg Fe/mL) for 1 day of 5 days or in the presence of a combination of FAC (4 µg Fe/mL) and NAC (4mM) for 5 days (n = 3-4). Data are presented as luminescence units. **(C-F)** Each dot represents an individual donor. One-way ANOVA with Dunnett’s multiple comparisons test was performed. *p < 0.05, **p < 0.01.

To understand the molecular basis of iron-induced inhibition of macrophage differentiation, we performed transcriptome profiling of monocytes differentiated with M-CSF in the absence or presence of FAC for 6 and 72 hours. Consistent with their surface phenotype, MDMs differentiated in the presence of FAC failed to elicit MDM core genes, including *MERTK*, *MRC1* (encoding CD206), and *TFRC* (encoding CD71) (**Figures 5A**, **S5A**). Moreover, transcriptional pathway analysis identified the NRF2-mediated oxidative stress response as being upregulated in MDM differentiated in the presence of FAC (**Figures 2A**, **S5B**). FAC exposure increased expression of multiple NRF2-dependent genes (e.g. *HMOX1*, *GCLM*, and *NQO1*) early during differentiation, reaching their peak expression after 6h and 24h, respectively (**Figure 2B**). We therefore assessed cellular ROS production in MDM differentiated in the presence of FAC. At day 5 of culture, increase of cellular ROS was observed in several donors and the ROS signal was localized to the nucleus and mitochondria as detected by CellROX Green dye (**Figure 2C**). In contrast, presence of sRBC during MDM differentiation neither induced NRF-2 dependent gene expression (**Figure 2B**), nor an immature phenotype or function (**Figure 1**). Hence the treatment with sRBC was excluded from further mechanistic analyses.

**Figure 3:**
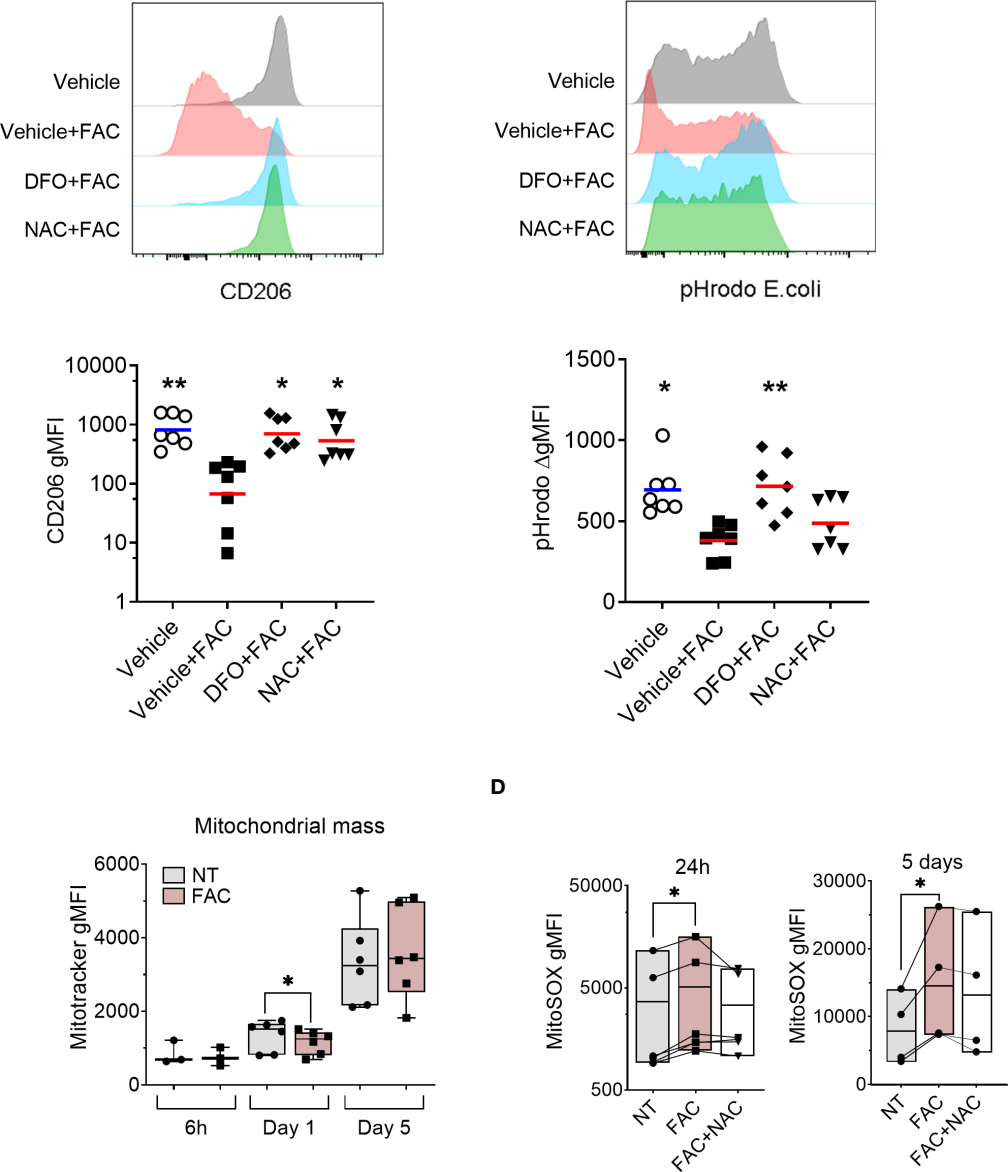
FAC-induced defective monocyte to macrophage differentiation is partially rescued by the antioxidant NAC. **(A)** Surface expression of CD206 on macrophages differentiated with M-CSF for 3 days in the presence of FAC (4 µg Fe/mL), deferoxamine mesylate salt (DFO, 87.5 μM), NAC (4mM), or vehicle 0.4% DMSO. **(B)** Phagocytic capacity of macrophages differentiated with M-CSF in the presence of FAC (4 µg Fe/mL), DFO (87.5μM), NAC (4mM), or vehicle 0.4% DMSO. Data are shown as the increment in fluorescence intensity of engulfed pHrodo *E. coli* bioparticles between cells incubated at 37°C and at 4°C. Each dot represents an individual donor (n = 7-8). Data shown are pooled from two experiments. One-way ANOVA with Dunnett’s multiple comparisons test was performed. *p < 0.05, **p < 0.01. **(C)** Quantification of mitochondrial mass in monocytes differentiated with M-CSF in the presence or absence of FAC (4µg Fe/mL) for 6h, 1 day and 5 days, in comparison to cells differentiated with M-CSF alone and presented as Mitotracker gMFI (n = 3-6). **(D)** Quantification of mtROS production in monocytes differentiated with M-CSF and treated with FAC (4µg Fe/mL) or a combination of FAC (4µg Fe/mL) and NAC (4mM) for 24h (n = 6) and 5 days (n = 4). Data are presented as MitoSOX gMFI. Each symbol represents an individual donor. One-way ANOVA with Dunnett’s multiple comparisons test was performed. *p < 0.05.

Given that mitochondria represent one of the primary sources of ROS (40) and are the main consumers of intracellular iron (41), we assessed the mitochondrial ROS (mtROS) production and mitochondrial function in MDM in the presence of FAC. We observed that mtROS production increased as the differentiation of monocytes progressed (**Figure 2C**). Additionally, FAC induced significantly higher mtROS production after 24h of treatment. A similar trend was observed after 6h and 5 days of treatment (**Figure 2C**). Considering that excessive mtROS production can damage mitochondria (42), we next analyzed the effects of FAC on mitochondrial health. On day 5 of differentiation, FAC treatment reduced mitochondria basal oxygen consumption rate (OCR) and maximal respiration OCR (**Figure 2D**), as well as mitochondrial membrane potential (mΔψ) (**Figure 2E**) and ATP production (**Figure 2F**), indicating impaired mitochondrial function. Despite a small decrease of mitochondrial mass, FAC did not induce a decrease of mΔψ or ATP production after 1 day of treatment (**Figures 2E-F**, **3C**). Importantly, the ROS scavenger N-acetyl cysteine (NAC) (**Figure 3D**) was able to partially revert the FAC-induced effects on mitochondrial respiration, mΔψ and ATP production (**Figures 2D-F**), supporting the notion that FAC-induced oxidative stress impairs mitochondrial function.

### Iron chelator and antioxidant treatments alleviate iron-induced defective macrophage differentiation

Next, we tested whether the observed effects of FAC on ROS production and mitochondrial function are linked to impaired MDM differentiation using the ROS scavenger NAC. We also employed the free iron chelator deferoxamine (DFO) as a positive control. As expected, iron chelation with DFO restored macrophage differentiation as evidenced by the rescued surface expression of CD206 (**Figure 3A**) and phagocytic capacity (**Figure 3B**). Importantly, scavenging ROS with NAC improved macrophage differentiation as demonstrated by restored CD206 surface expression and partial recovery of phagocytic capacity (**Figures 3A, B**).

**Figure 4:**
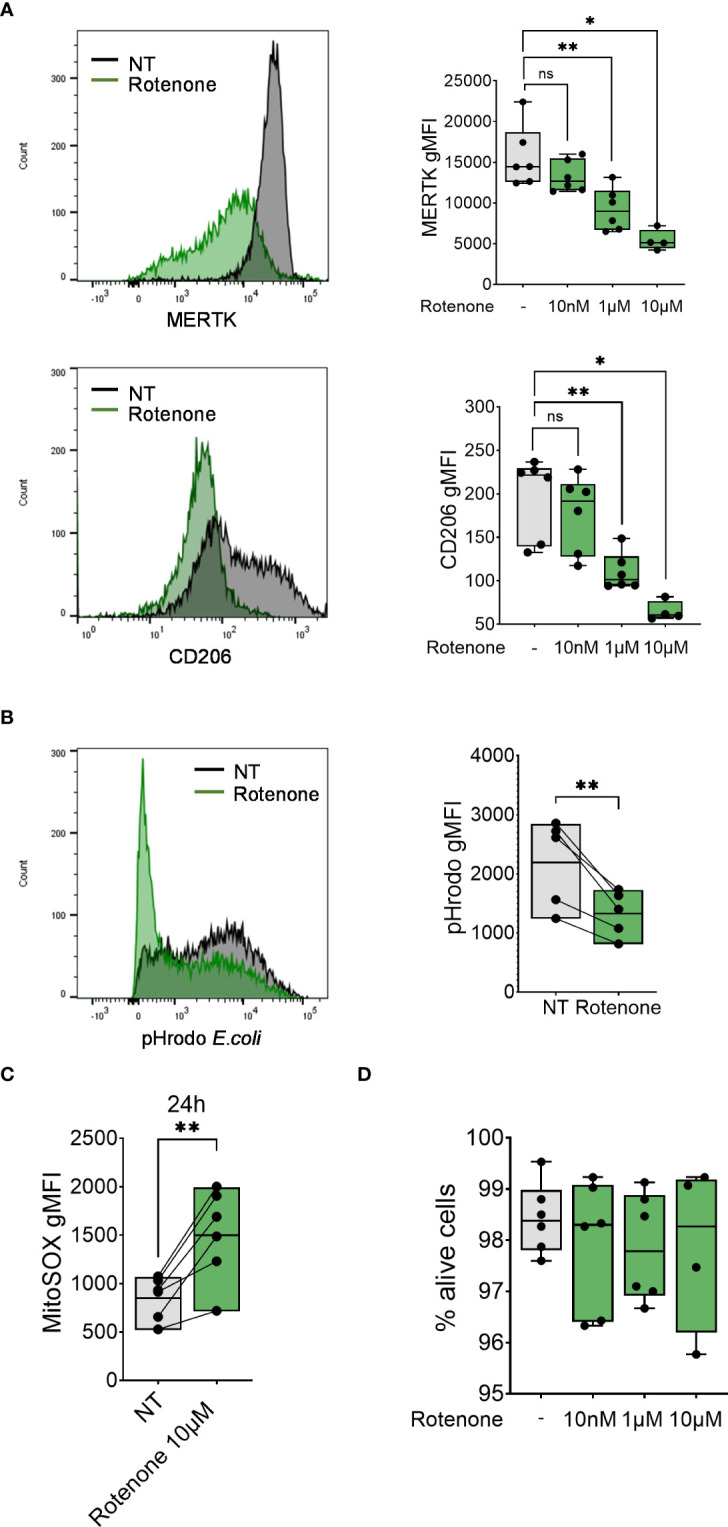
Rotenone induces mtROS production and impairment of monocyte-to-macrophage differentiation. **(A)** Surface expression of indicated markers analyzed on monocyte differentiated with M-CSF in the presence of rotenone (10nM-10µM) for 1 day. Each symbol represents an individual donor (n = 4-6). Data shown are pooled from three experiments. **(B)** Phagocytic capacity of monocytes differentiated with M-CSF in the presence of 10µM rotenone for 1 day, represented as pHrodo *E.coli* bioparticle gMFI. Each dot represents an individual donor (n = 6). Data shown are pooled from three experiments. **(C)** Quantification of mtROS production in monocytes differentiated with M-CSF and treated with rotenone 10µM for 24h (n = 6). Data are presented as MitoSOX gMFI. **(D)** FACS analysis of the percentage of viable monocytes (taken as Zombie Green negative cells) differentiated with M-CSF and treated with rotenone for 1 day. Each symbol represents an individual donor. **(A-C)** One-way ANOVA with Dunnett’s multiple comparisons test was performed. ns, not significant, *p < 0.05, **p < 0.01.

To further assess the contribution of mtROS to macrophage differentiation, we analyzed MDM markers (CD206 and MERTK surface expression) and function (phagocytosis of pHrodo-coated *E.coli* bioparticles) in differentiating monocytes after treatment with the mtROS inducer rotenone (43) (**Figure 4C**). Treatment with rotenone reduced surface expression of both markers MERTK and CD206 in a dose-dependent manner (**Figure 4A**) and decreased monocytes’ phagocytic capacity (**Figure 4B**) without altering cell viability (**Figure 4D**), suggesting that the induction of mtROS is sufficient to impair monocyte-to-macrophage differentiation.

### FAC impairs macrophage differentiation by inhibiting M-CSF-induced upregulation of MAFB and M-CSF receptor

**Figure 5:**
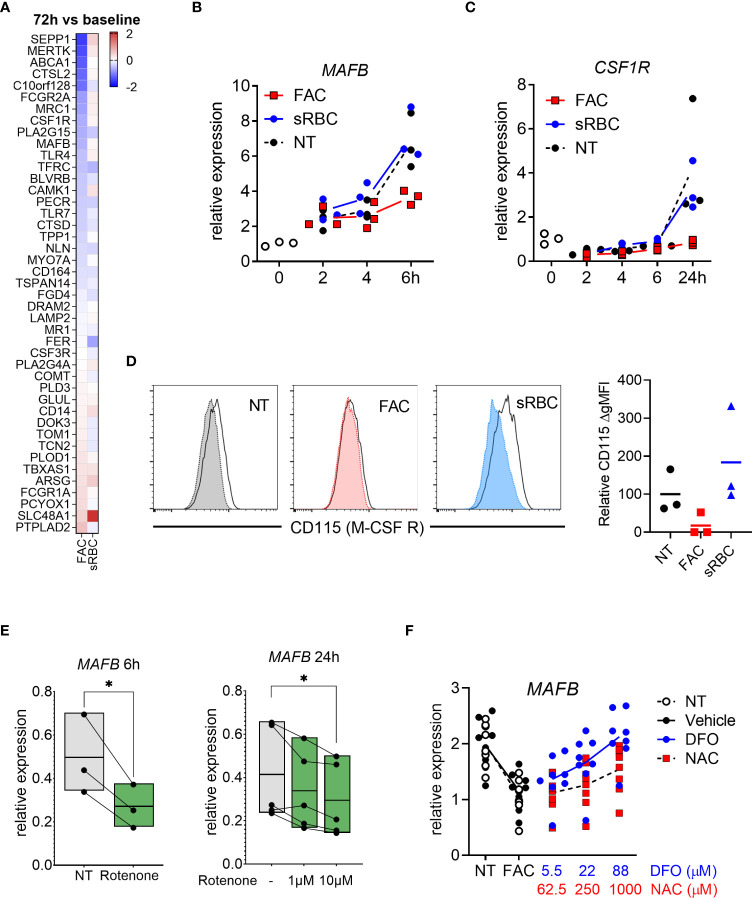
Iron overload-induced inhibition of monocyte-to-macrophage differentiation is mediated by MAFB. **(A)** Heatmap depicting Log2 fold change of human macrophage signature genes (46) in monocytes differentiated with M-CSF in the presence of FAC (4 µg Fe/mL) or sRBC for 72h compared to cells treated with only M-CSF. **(B, C)** mRNA expression relative to 0h of *MAFB* and *CSF1R* (encoding M-CSF receptor) in monocytes differentiated with M-CSF in the presence or absence of FAC (4µg Fe/mL) or sRBC for 0-24h (n = 3). **(D)** Surface expression of M-CSF receptor (CD115) on monocytes differentiated with M-CSF in the presence or absence of FAC (4µg Fe/mL) or sRBC for 3 days (n = 3). Shaded histograms show fluorescence minus one (FMO) controls. **(E)** Normalized mRNA expression of *MAFB* in monocytes differentiated with M-CSF in the presence of 10µM rotenone for 6h or 1µM to 10 µM for 24h (n = 3-5). **(F)** mRNA expression relative to 0h of *MAFB* in monocytes differentiated with M-CSF and treated with FAC (4µg Fe/mL) in combination with DFO or NAC for 3 days. Vehicle control is 0.4% DMSO (n = 8). **(B-F)** Each dot represents an individual donor. One-way ANOVA with Dunnett’s multiple comparisons test was performed. ns, not significant, *p < 0.05.

To better understand the mechanistic link between oxidative stress and macrophage differentiation, we revisited the transcriptomic data. We noticed that gene expression of the transcription factor MAFB and M-CSF receptor (*CSF1R*), both of which are crucial for M-CSF-induced macrophage differentiation (44, 45), was inhibited by FAC after 72 hours of treatment (**Figure 5A**). We confirmed by RT-PCR that the decrease in *MAFB* and *CSFR1* gene expression caused by FAC started from 6 and 24 hours of treatment, respectively (**Figures 5B, C**). Consistently, M-CSF-induced surface expression of M-CSF receptor (also known as M-CSFR or CD115) was also prevented at day 3 of culture in the presence of FAC (**Figure 5D**). However, sRBC did not alter *MAFB* or *CSFR1* gene expression or M-CSFR surface expression in differentiating monocytes (**Figures 5B-D**). In addition, rotenone induction of mtROS also resulted in decreased *MAFB* gene expression compared to untreated monocytes after 6 and 24 hours of treatment in a dose-dependent manner (**Figure 5E**). Notably, the extent of the effects of rotenone on monocyte-to-macrophages differentiation was comparable to those caused by FAC (**Figures 1**, **S2**).

Importantly, NAC treatment restored *MAFB* gene expression to a level comparable to that of MDM differentiated in the absence of FAC or to monocytes treated with FAC in the presence of DFO (**Figure 5F**).

Together, these data indicate that FAC-mediated induction of mtROS may be sufficient to impair monocyte-to-macrophage differentiation by inhibiting *MAFB* and *CSF1R* gene expression.

## Discussion

Macrophages play a crucial role in the maintenance of tissue homeostasis and tissue repair beyond their well-known role in the elimination of invading pathogens and coordination of the immune response. Defective macrophage differentiation from circulating monocytes impairs the formation of fully functional macrophages and therefore leads to detrimental effects in pathogen elimination and tissue homeostasis (47–49). How the tissue microenvironment interferes with macrophage differentiation is incompletely understood.

Here we have developed an iron-overload *in vitro* model to demonstrate how an excess of exogenous iron impairs successful differentiation of monocytes into functional macrophages. Ferric iron (Fe^3+^) is the stable iron oxidation state under aerobic conditions, and in complex with transferrin it is the major non-heme form of iron used by cells (50). FAC, which is a source of ferric iron and therefore a relevant *in vivo* correlate for transferrin-bound non-heme iron, prevented M-CSF-stimulated upregulation of the macrophage maturation surface markers MERTK (37), CD206 (38), and CD71 (39), involved in immune functions such as efferocytosis, pathogen pattern recognition and transferrin uptake, both on a protein and transcriptional level. Excessive iron also negatively affected the functional capacity of macrophages to phagocytize bacterial particles. These results are in accordance with a report from Fell et al (51) in which the exposure of differentiating primary monocytes to several iron preparations used for intravenous transfusion decreased macrophage phagocytic capacity. In contrast, heme iron (sRBC) did not impair macrophage differentiation. Iron overload by sRBC is a complex process, and unlike FAC uptake *via* iron transporters, the process of sRBC requires phagocytosis in which sRBC are sequestered in the phagolysosomes and undergo enzymaticc degradation to release heme. After transport into the cytosol, heme is degraded by heme oxygenase resulting in the release of iron, which can be stored in ferritin or exported out of the cell by ferroportin (FPN1) (13, 52). Unlike FAC, sRBC induce monocyte differentiation toward an iron recycling macrophage phenotype with an increased expression of iron exporter FPN1, which may explain a lower level of iron accumulation in the cells. Despite iron accumulation, sRBC did not alter the expression of MDM maturation markers or phagocytic activity, suggesting that exposure to sRBC was not sufficient to achieve the iron levels required to impair differentiation to macrophages. Alternatively, additional iron-processing mechanisms not investigated in this manuscript are in place. Interestingly, it has been recently published (53) that treatment of human lung macrophages or fully differentiated MDMs with hemin, decreases gene expression of M2 macrophage markers and *FPN*, macrophage phagocytic capacity and cytokine secretion. However, this same study did not use stressed RBCs nor found an effect of FAC on macrophage parameters when using a similar or even double the FAC concentrations we used in this study. These results suggest that, not only the type of iron (hemin, sRBC or free iron) has different effects on macrophage function, but also whether the iron stimulation happens in monocytes that are in the process of differentiation or are fully differentiated into macrophages may be important.

Macrophage dysfunction, including defective phagocytosis of bacterial pathogens and defective clearance of apoptotic cells by efferocytosis, has consistently been associated with COPD (54). It has been proposed that macrophages from COPD patients do not fit into the conventional phenotypes of macrophage differentiation, suggesting the existence of a specific COPD macrophage phenotype (55). There is also strong evidence pointing to a link between iron dysregulation and COPD based on human genetics (56), murine studies (28), and observations of increased iron deposition in COPD lung tissue (30) and alveolar macrophages (29, 57). One common feature related to macrophage dysfunction, iron overload and COPD may be exposure to cigarette smoke. Cigarette smoke exposure impairs monocyte differentiation into macrophages (58, 59), and previous observations suggest that cigarette smoke may be a significant exogenous source of iron (60) and that it may increase iron deposition systemically and in alveolar macrophages from smokers and COPD patients (29, 61, 62). Still, direct evidence that iron is responsible for the defective differentiation of monocytes caused by the exposure to cigarette smoke is still lacking.

Pathway analysis of the transcriptional changes induced by iron overload during macrophage differentiation revealed an induction of the cellular response to oxidative stress, especially genes regulated by NRF2, which plays an important role against oxidative stress in macrophages (63). Flow cytometry analysis showed that while the production of mitochondrial reactive oxygen species (mtROS) progressively increased during macrophage differentiation, exposure of iron induced an additional increase in mtROS production. mtROS (mainly superoxide anion) are normal byproducts of mitochondrial respiration and play important roles in regulating signaling pathways (64) and gene expression (65) in immune cells, and can even promote cell differentiation of stem cells (66), myoblasts (67), vascular smooth muscle cells (68),and adipocytes (69). Cellular ROS and mtROS have been found to be induced during the differentiation of monocytes into dendritic cells (DCs) with GM-CSF (70), but, to our knowledge, no evidence of mtROS production during monocyte differentiation into macrophages with M-CSF has been reported. Our investigations demonstrate that mtROS plays an important role in the differentiation of human monocytes into macrophages.

Proper function of mitochondria has been shown to be critical during differentiation of monocyte derived DC and required for repolarization of inflammatory macrophages in human and mice (71–73). However, the role of mitochondria in M-CSF-induced monocyte to macrophage differentiation has not been yet investigated. In our experiments, M-CSF-induced differentiation of monocytes also caused a gradual increase in mitochondrial mass, which could explain the parallel increase in mtROS production during monocyte differentiation. However, iron overload did not induce any additional increase in mitochondrial mass for any of the time points analyzed, suggesting an alternative mechanism behind the induction of excessive mtROS production.

As mentioned above, we observed a gradual increase in mitochondria mass and mtROS production during the process of monocyte differentiation into macrophages with M-CSF. These changes were accompanied by an increase in mitochondrial membrane potential (mtΔψ), which controls mitochondria respiratory rate and ATP production (74). However, the addition of excessive iron caused a drop in mtΔψ after 5 days of differentiation. In line with this, we observed defective mitochondria metabolism in iron-treated cells, as evident from the reduction in basal oxygen consumption rate, maximal respiration rate and ATP production.

Interestingly, the increase of mtROS production in iron overload monocytes correlates in time with the induction of the differentiation defects, pointing at mtROS as a possible cause behind the iron-induced differentiation defects. In contrast, the iron-induced mitochondrial damage occurs at much later time point than the observed defects on monocyte differentiation, suggesting that the decreased mitochondria activity is not the ultimate cause of the impaired macrophage differentiation upon iron treatment but a parallel event.

Supporting the notion that iron-induced increase of mtROS production is a significant contributor to the mitochondrial damage and macrophage differentiation, our results show that the ROS scavenger NAC partially rescued the defects in OCR, ATP, and mitochondria membrane potential. These findings are supported by literature in macrophages (75) and in other cell types (76).

Although a certain amount of mtROS is induced during monocyte to macrophage differentiation, our results indicate that the excessive production of mtROS is detrimental to functional macrophage differentiation. The iron-induced defects in CD206 expression and phagocytic capacity are at least partially rescued by treatment with the ROS scavenger NAC. The incomplete rescue of macrophage differentiation by NAC might be due to other involved unknown mechanisms or by ineffective quenching of mtROS by NAC.

To further support our hypothesis that mtROS is the major contributor to defective macrophage differentiation, we applied an alternative mtROS inducer, rotenone, during M-CSF-induced monocyte-to-macrophage differentation. Similar to what we observed with FAC treatment, rotenone treatment to monocytes was sufficient to decrease the surface expression of MERTK and CD206 and the capacity of macrophages to phagocytize bacteria particles.

Mechanistically, our transcriptomic analysis of core macrophage genes revealed that treatment with FAC decreased gene expression of *MAFB*, a master transcription factor that regulates differentiation of monocytes into macrophages (44, 77, 78), and of the M-CSF receptor (*CSF1R*) in differentiating monocytes. Similarly, rotenone also inhibits MAFB upregulation during macrophage differentitaion. Moreover, ROS scavenging with NAC rescued FAC-induced inhibition of *MAFB* gene expression strongly suggesting a role of ROS in the regulation of *MAFB*. To our knowledge, there are no reports demonstrating a direct role of iron or ROS production in the regulation of *MAFB* gene expression. However, other transcription factors regulating cell differentiation have been reported to be influenced by ROS, for example, NFkB and STAT5, involved in the differentiation of monocytes into dendritic cells and macrophages, respectively (79–82). How exactly iron or ROS production regulate *MAFB* gene expression may be the object of future investigations.

Taken together, our results show how non-heme iron overload impairs M-CSF-induced monocyte-to-macrophage differentiation resulting in mitochondrial damage and excessive mtROS production. We also demonstrate how mtROS might be an important contributing factor in the regulation of monocyte differentiation into macrophages, possibly *via* MAFB.

## Data availability statement

RNA-sequencing data underlying the findings described in this manuscript may be obtained in accordance with AstraZeneca’s data sharing policy described at https://astrazenecagrouptrials.pharmacm.com/ST/Submission/Disclosure. Use the “Enquiries about Vivli Member Studies” (https://vivli.org/members/enquiries-about-studies-not-listed-on-the-vivli-platform/) form and include the publication title and data accession number GSF765277 in your request.

## Author contributions

YC, SG, UG, SC, TN and HO contributed to the conception and design of the study. YC, SG, SA and KT performed the experiments. YC, SG, SA and LÖ analyzed the data. YC and SG wrote the first draft of the manuscript. YC, SG, UG, SC, TN and HO contributed with specific sections and the revision of the manuscript. All authors contributed to the article and approved the submitted version.

## Funding

SC is supported by Science Foundation Ireland Future Research Leaders Grant FRL4862.

## Acknowledgments

We are grateful to volunteers who contributed with blood samples toward this study. We also thank Katarina Vesterlund for blood collection; Shalini Venkatesan, Xiao-Hong Zhou, Melker Göransson, and Neda Najafinobar for their scientific input and technical support. SG and YC are current & alumni fellows of the AstraZeneca postdoc program.

## Conflict of interest

SG, SA, LÖ, KT, UG, TN, HO, and YC are current or former employees of AstraZeneca. YC is an employee of SangamoTherapeutics.

The remaining author declares that the research wasconducted in the absence of any commercial or financialrelationships that could be construed as a potential conflictof interest.

## Publisher’s note

All claims expressed in this article are solely those of the authors and do not necessarily represent those of their affiliated organizations, or those of the publisher, the editors and the reviewers. Any product that may be evaluated in this article, or claim that may be made by its manufacturer, is not guaranteed or endorsed by the publisher.
