# Supplementary material for: Non-heme iron overload impairs monocyte to macrophage differentiation via mitochondrial oxidative stress
Source: Front Immunol. 2022 Oct 21;13:998059. doi: 10.3389/fimmu.2022.998059 (PMC9634638; doi:10.3389/fimmu.2022.998059)
Supplement: Supplementary file 1 [file DataSheet_1.pdf]

Figure S1

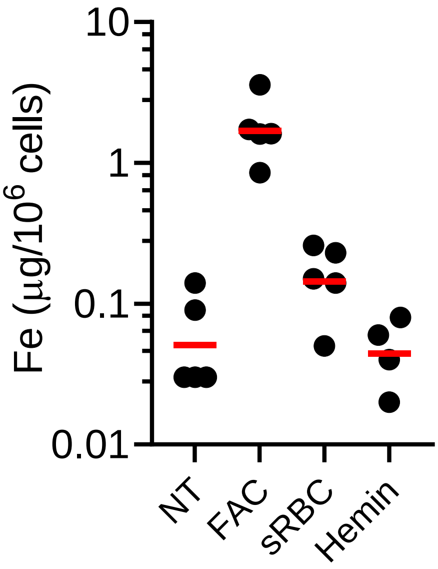

Supplementary Figure S1. Iron accumulation during monocyte-to-macrophage differentiation. Cellular iron was quantified using inductively coupled plasma-mass spectrometry. All cultures were analyzed 3-5 days after the respective treatment in the presence of M-CSF. Ferric ammonium citrate (FAC, 4 µg Fe/mL, 86.8 µM*)*, stressed RBC (sRBC, 10x monocytes), hemin (40-50 µM), no additional iron (NT). Each symbol represents an individual blood donor (n=4-5).

Figure S2

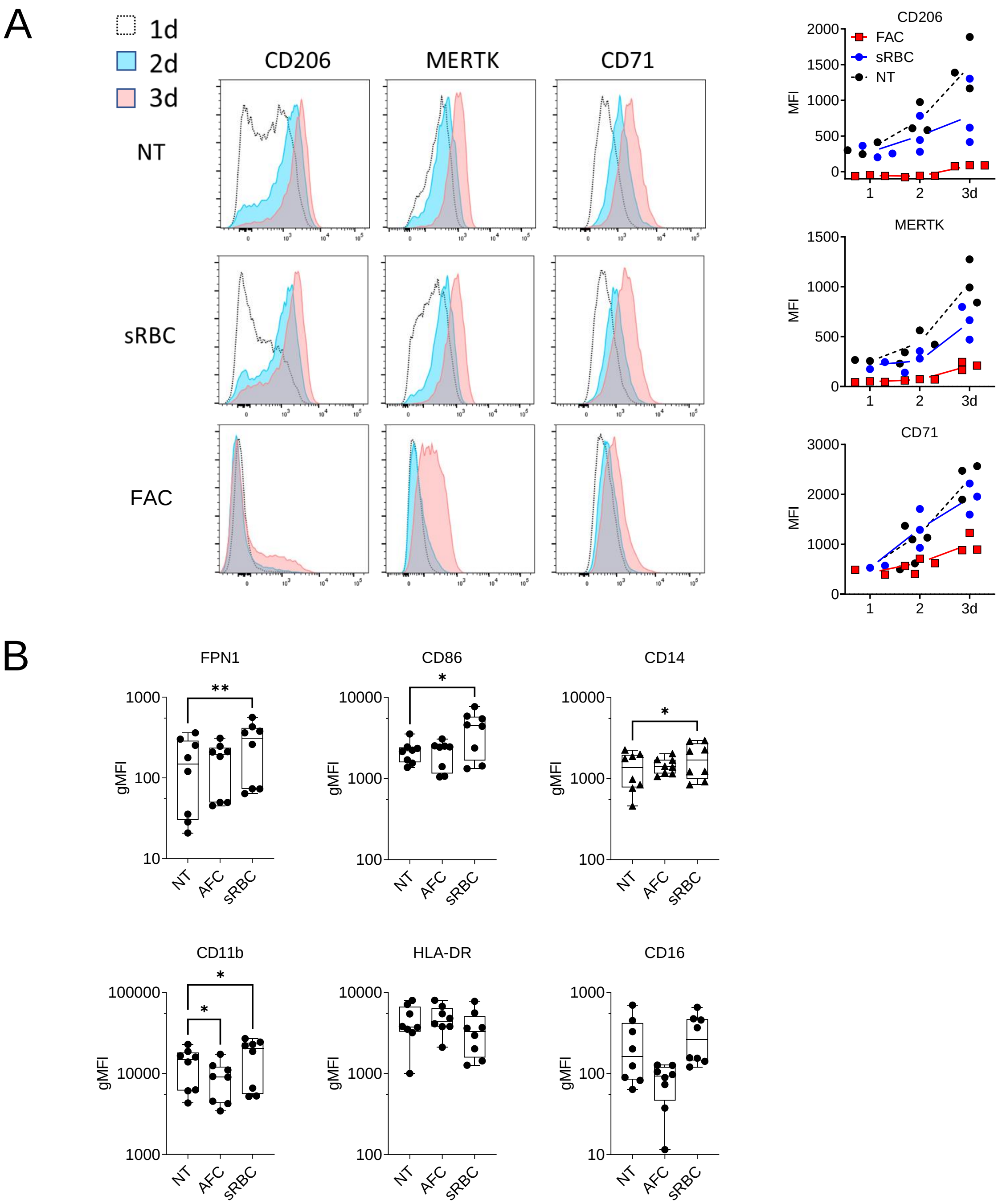

Figure S2. FAC and sRBC modulate surface phenotypic markers on monocyte-derived macrophages differentiated with M-CSF.

(A) Analysis of surface markers on monocytes cultured with M-CSF and FAC (4 Fe µg/mL) or sRBC (10x monocytes) for 1-3 days compared to monocytes differentiated with M-CSF alone (NT) (n=3). (B) Surface expression of indicated markers analyzed on monocyte-derived macrophages after 5 days of differentiation with M-CSF in the presence of FAC (4 µg Fe/mL) or sRBC (10x monocytes) compared to monocytes differentiated with M-CSF alone (NT) (n=8). Each symbol represents an individual donor. Data shown are pooled from three experiments. One-way ANOVA with Dunnett's multiple comparisons test was performed. *$p < 0.05$, **$p < 0.01$.

Figure S3

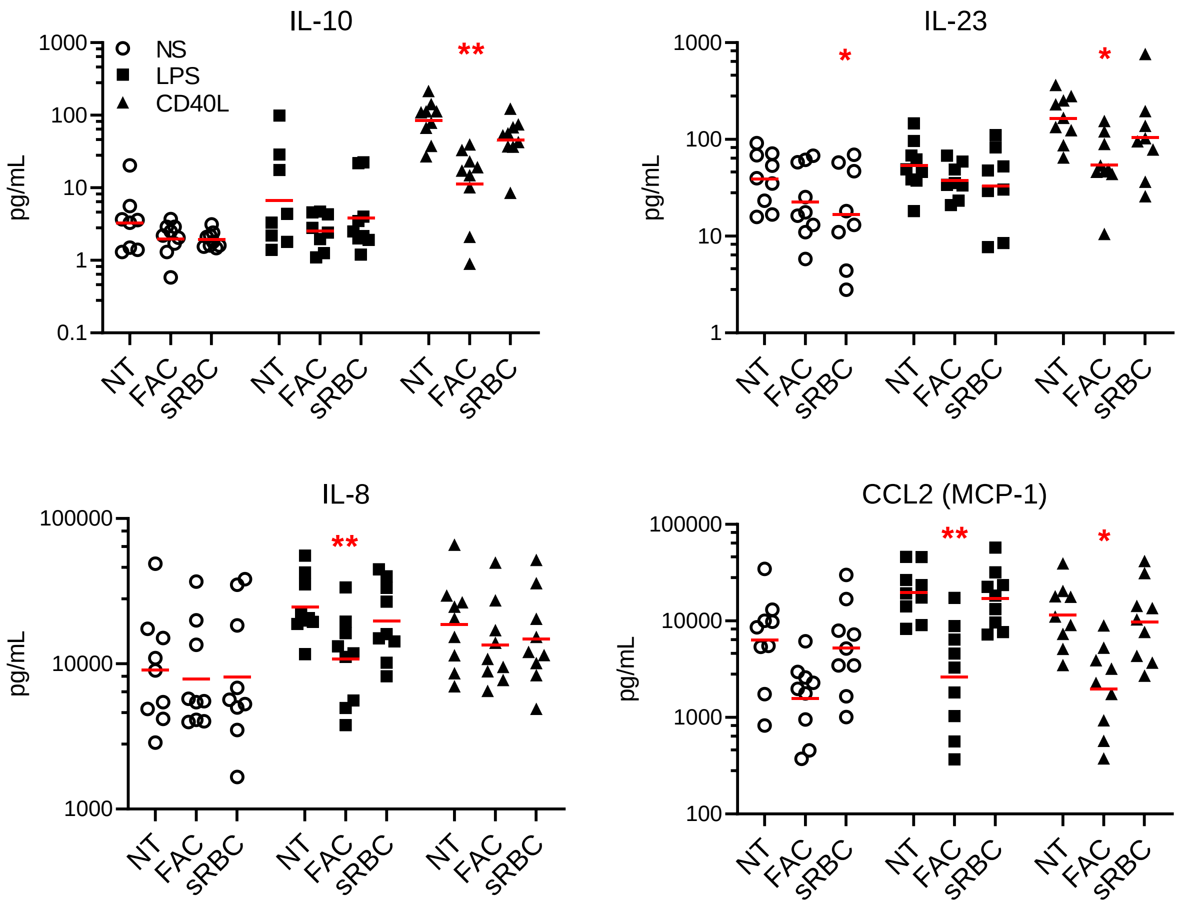

Figure S3. Reduced cytokine or chemokine production by MDMs differentiated in the presence of FAC.

Monocytes were differentiated with M-CSF in the presence or absence of FAC or sRBC for 6 days prior to stimulation with LPS or CD40L for 24h. Supernatants were collected and cytokine/chemokine secretion were quantified using MSD. Each symbol represents an individual donor. Results represent a pool of 9 donors. Two-way ANOVA with Dunnett's multiple comparisons test was performed. *$p < 0.05$, **$p < 0.01$

Figure S4

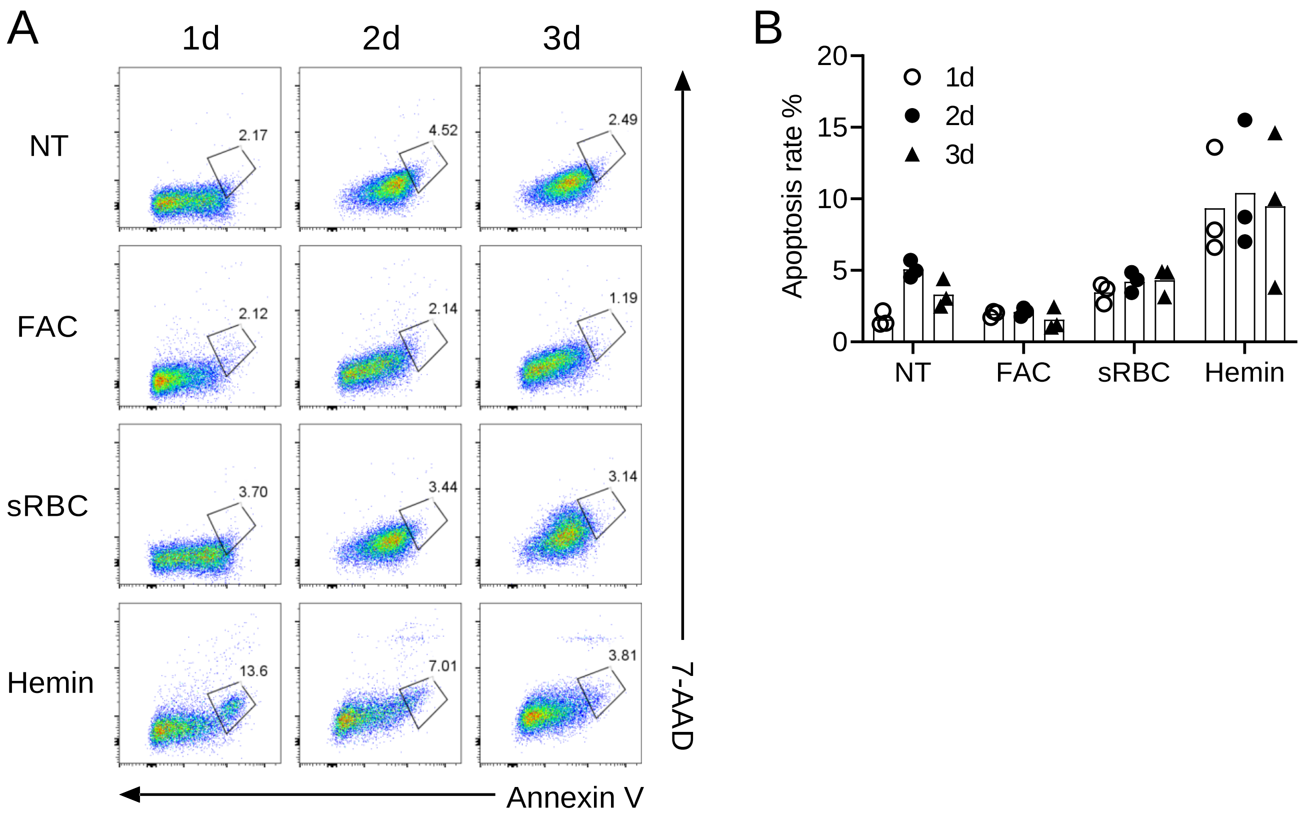

Figure S4. FAC-induced defective monocyte-to-macrophage differentiation was not a result of apoptosis.

(A, B) Apoptotic cells were detected with Annexin V and 7-AAD staining from monocytes cultured with M-CSF and FAC (4 µg Fe/mL), sRBC (10x monocytes) or Hemin (100 µM) for 1-3 days. Monocytes differentiated with M-CSF alone were used as controls (NT) (n=3). Each symbol represents an individual donor.

Figure S5

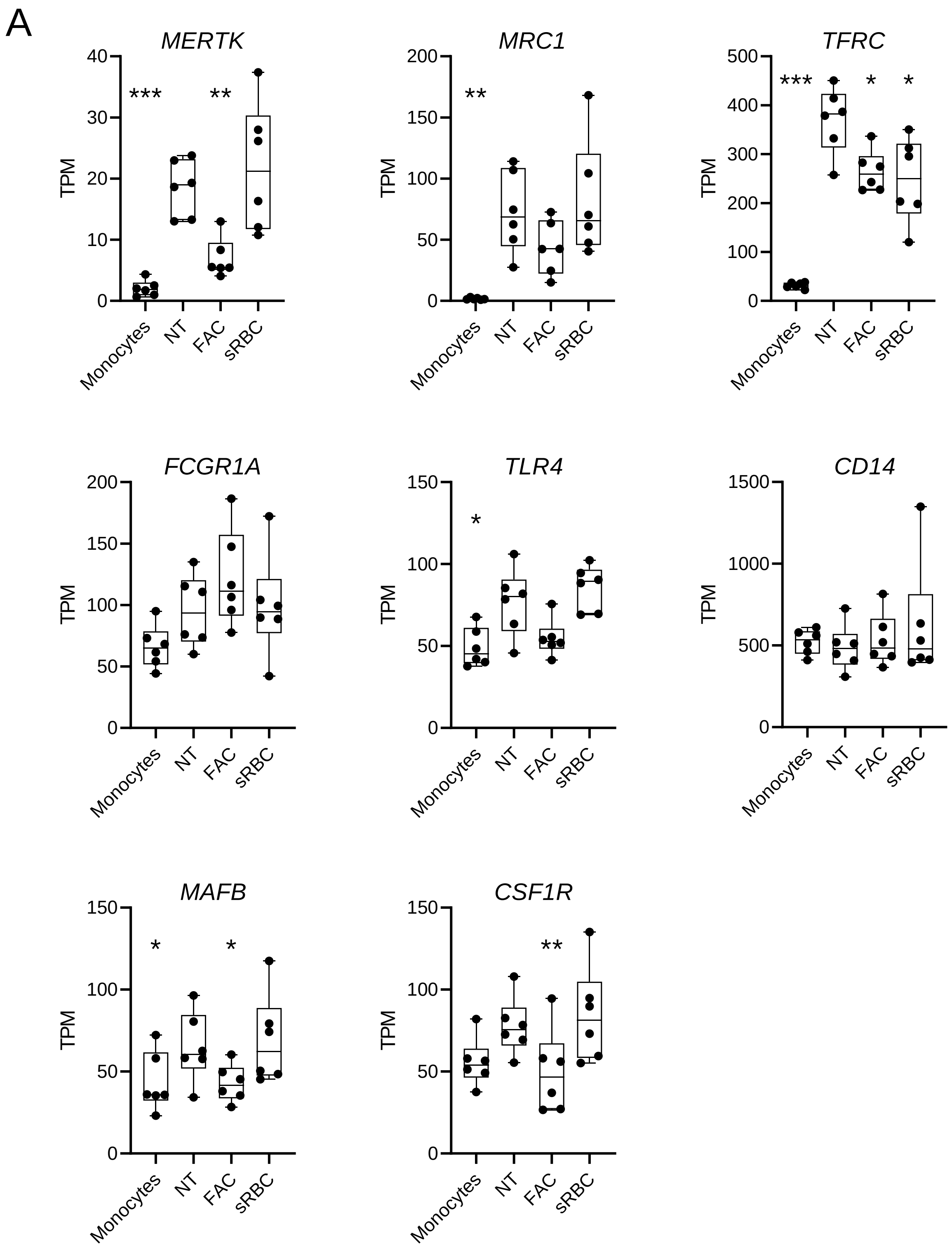
A
MERTK
MRC1
TFRC
FCGR1A
TLR4
CD14
MAFB
CSF1R
TPM
Monocytes
NT
FAC
sRBC
***
**
*
0
10
20
30
40
50
100
150
200
300
400
500
1000
1500

Figure S5 (continued)

B

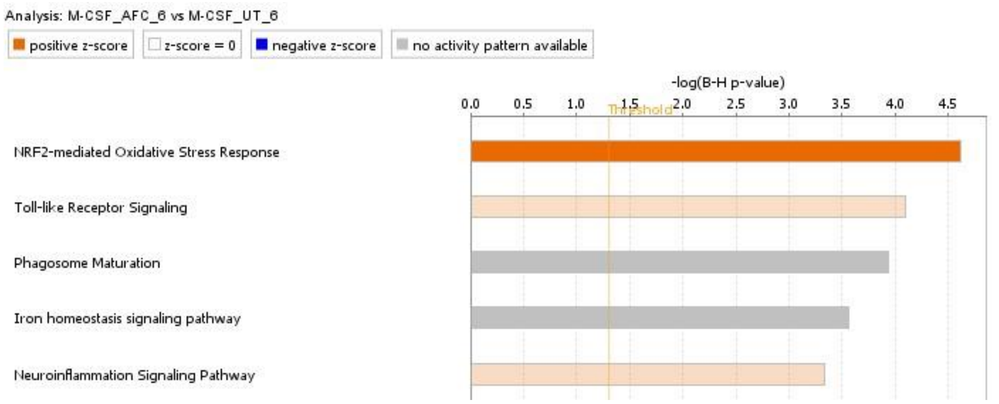

C

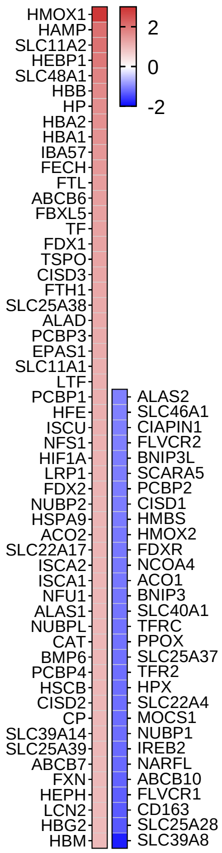

D

Top Upstream Regulators

Upstream Regulators

Name	p-value	Predicted Activation
**IFNG**	3.46E-16	
**MAFB**	5.27E-11	Inhibited
**TLR7**	1.36E-08	Inhibited
**LDLR**	1.70E-08	
**TNF**	3.68E-08	

Top Regulator Effect Networks

ID	Regulators	Disease & Functions	Consistency Score
1	CSF1,MAFB,mir-155,TLR3	Engulfment of cells (+4 more)	7.826
2	TLR3,TLR7	Migration of dendritic cells (+1 more)	4.243
3	MYD88,STAT3,TLR7	Migration of dendritic cells	4.025
4	STAT1	Leukopoiesis	1.225
5	CSF1	Hematopoiesis of mononuclear leukocytes	-2.846

Figure S5 (continued)

E

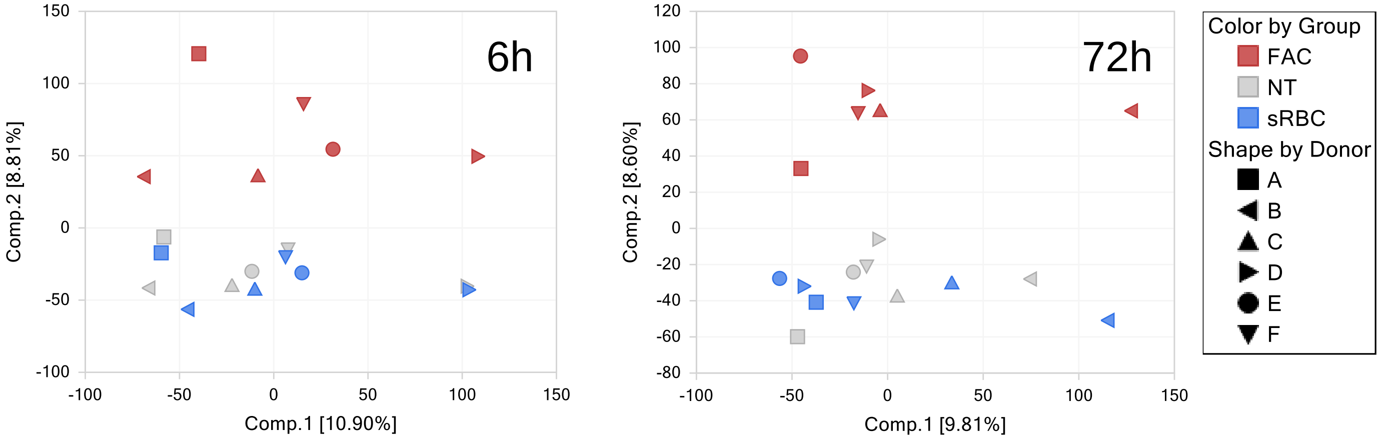

F

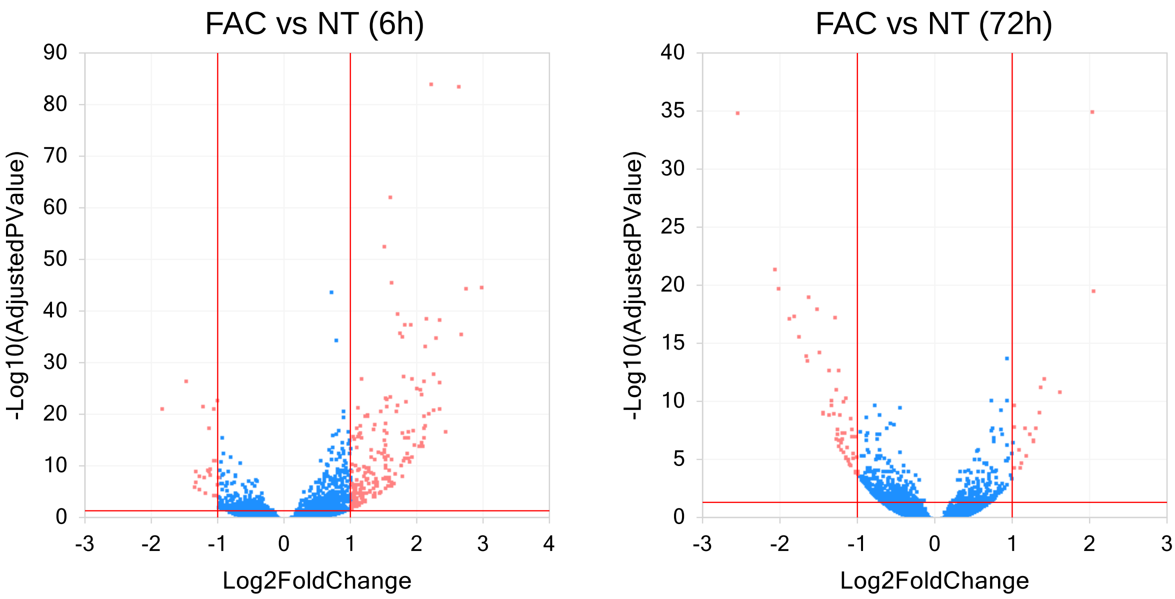

Figure S5. NRF2 oxidative stress response, transcription factor MAFB, and M-CSFR regulate FAC-induced defective monocyte-to-macrophage differentiation

(A) Gene expression (TPM, transcripts per million) of representative human macrophage signature genes (ref. 46) in monocytes differentiated with M-CSF in the presence or absence of FAC (4 µg Fe/mL) or sRBC for 72h. (B-D) Transcriptional pathway analysis of monocytes differentiated with M-CSF in the presence or absence of FAC (4 µg Fe/mL) for 6 or 72h using Ingenuity Pathway Analysis application. (B) Table with the top 5 most statistically significant canonical pathways regulated by FAC after 6h treatment. (C) Heatmap depicting Log2 fold change of iron-related genes in monocytes cultured with M-CSF and FAC (4 µg Fe/mL) for 6h compared to monocytes cultured with M-CSF alone. (D) Table with the top upstream regulators or top regulator effect networks regulated by FAC after 72h treatment. (E) Principal component analysis (PCA) of all genes showed clustering by donor and treatment. (F) Volcano plots highlighting in red genes significantly regulated by FAC treatment at 6h (216 genes) and 72h (72 genes), log2-fold-change > 1, adjusted pvalue < 0.05.
